# Nivolumab Plus 5-Azacitidine in Pediatric Relapsed/Refractory Acute Myeloid Leukemia (AML): Phase I/II Trial Results from the Therapeutic Advances in Childhood Leukemia and Lymphoma (TACL) Consortium

**DOI:** 10.3390/cancers16030496

**Published:** 2024-01-24

**Authors:** Anupam Verma, Yueh-Yun Chi, Jemily Malvar, Adam Lamble, Sonali Chaudhury, Archana Agarwal, Hong-Tao Li, Gangning Liang, Roy Leong, Patrick A. Brown, Joel Kaplan, Eric S. Schafer, Tamra Slone, Melinda Pauly, Bill H. Chang, Elliot Stieglitz, Alan S. Wayne, Nobuko Hijiya, Deepa Bhojwani

**Affiliations:** 1 Center for Cancer and Blood Disorders, Pediatric Hematology Oncology Branch, Children’s National Hospital, Washington, DC 20010, USA; 2 Division of Pediatric Hematology Oncology, Primary Children’s Hospital, University of Utah, Salt Lake City, UT 84113, USA; 3 Division of Hematology-Oncology, Cancer and Blood Disease Institute, Children’s Hospital Los Angeles, Norris Comprehensive Cancer Center, Keck School of Medicine, University of Southern California, Los Angeles, CA 90027, USA; ychi@chla.usc.edu (Y.-Y.C.); awayne@chla.usc.edu (A.S.W.); dbhojwani@chla.usc.edu (D.B.); 4 Division of Hematology-Oncology, Cancer and Blood Disease Institute, Children’s Hospital Los Angeles, Los Angeles, CA 90027, USA; jmalvar@chla.usc.edu (J.M.);; 5 Department of Pediatric Hematology Oncology, Seattle Children’s Hospital, Seattle, WA 98105, USA; adam.lamble@seattlechildrens.org; 6 Department of Pediatric Hematology Oncology, Ann and Robert Lurie Children’s Hospital of Chicago, Chicago, IL 60611, USA; schaudhury@luriechildrens.org; 7 Department of Pathology, University of Utah and ARUP Laboratories, Salt Lake City, UT 84108, USA; archana.agarwal@aruplab.com; 8 Department of Urology, University of Southern California, Los Angeles, CA 90033, USA; hongtaol@usc.edu (H.-T.L.); gliang@usc.edu (G.L.); 9 Bristol Myers Squibb, Princeton, NJ 08543, USA; 10 Department of Pediatric Hematology Oncology, Atrium Health Levine Children’s Hospital, Wake Forrest University, Charlotte, NC 28203, USA; joel.kaplan@carolinashealthcare.org; 11 Division of Pediatric Hematology/Oncology, Baylor College of Medicine, Texas Children’s Cancer and Hematology Center, Houston, TX 77030, USA; esschafe@txch.org; 12 Department of Pediatric Hematology Oncology, UT Southwestern, Dallas, TX 75235, USA; tamra.slone@utsouthwestern.edu; 13 Department of Pediatric Hematology Oncology, Children’s Healthcare of Atlanta, Atlanta, GA 30322, USA; melinda.pauly@choa.org; 14 Division of Pediatric Hematology Oncology, Oregon Health and Science University, Portland, OR 97239, USA; changb@ohsu.edu; 15 Department of Pediatric Oncology, University of California, San Francisco Benioff Children’s Hospitals, San Francisco, CA 94158, USA; elliot.stieglitz@ucsf.edu; 16 Division of Pediatric Hematology Oncology and Stem Cell Transplant, Columbia University Medical Center, New York, NY 10032, USA; nh2636@cumc.columbia.edu

**Keywords:** acute myeloid leukemia, immunotherapy, checkpoint inhibitor, DNA methylation, dose-limiting toxicity

## Abstract

**Simple Summary:**

The overall 5-year survival rate for children with acute myeloid leukemia (AML) has increased over time and is now in the range of 65% to 70%, but survival for patients who relapse continues to be poor. A phase I/II clinical trial was conducted by the pediatric consortium Therapeutic Advances in Childhood Leukemia & Lymphoma, with the aim to study immunotherapy along with azacitidine in children with relapsed AML. The combination was well tolerated in all the patients enrolled on the trial, with 33% having stable disease. In future studies, this combination in select pediatric patients with AML should be explored.

**Abstract:**

Improvements in survival have been made over the past two decades for childhood acute myeloid leukemia (AML), but the approximately 40% of patients who relapse continue to have poor outcomes. A combination of checkpoint-inhibitor nivolumab and azacitidine has demonstrated improvements in median survival in adults with AML. This phase I/II study with nivolumab and azacitidine in children with relapsed/refractory AML (NCT03825367) was conducted through the Therapeutic Advances in Childhood Leukemia & Lymphoma consortium. Thirteen patients, median age 13.7 years, were enrolled. Patients had refractory disease with multiple reinduction attempts. Twelve evaluable patients were treated at the recommended phase II dose (established at dose level 1, 3 mg/kg/dose). Four patients (33%) maintained stable disease. This combination was well tolerated, with no dose-limiting toxicities observed. Grade 3–4 adverse events (AEs) were primarily hematological. Febrile neutropenia was the most common AE ≥ grade 3. A trend to improved quality of life was noted. Increases in CD8+ T cells and reductions in CD4+/CD8+ T cells and demethylation were observed. The combination was well tolerated and had an acceptable safety profile in pediatric patients with relapsed/refractory AML. Future studies might explore this combination for the maintenance of remission in children with AML at high risk of relapse.

## 1. Introduction 

With current therapies, approximately 40% of children treated for newly diagnosed AML relapse. Despite great efforts by cooperative groups in the past several decades, the long-term survival rate of children with relapsed AML has not changed significantly and remains poor, with a probability of overall survival (pOS) of 16% to 34% [1,2]. The incorporation of novel agents into pediatric AML therapy has been limited and remains a high unmet need [3,4,5,6]. The intensification of conventional chemotherapy leads to increased toxicities without significant improvement in outcomes; therefore, novel therapeutic strategies are urgently needed. 

An improved understanding of the interaction between the immune milieu and tumor cells, and the role of immune evasion in tumor maintenance, has opened avenues for innovative immunotherapies. Programmed cell death-1 (PD-1) is an immuno-inhibitory receptor mainly expressed on activated T cells that plays a major role in tumor immune escape. Blockade of this pathway by anti-PD-1 antibodies allows T cells to maintain their antitumor functionality and ability to mediate tumor cell death [7,8]. Murine studies have shown that blockade of PD-1 and its ligand programmed cell death ligand-1(PD-L1) augments the induction, expansion, and survival of endogenous anti-leukemia T cells [9,10]. Nivolumab is a human IgG4 anti-PD-1 monoclonal antibody that has been studied as a single agent (NCT02275533) and in combination with chemotherapy (NCT02397720, NCT02464657) in adults with newly diagnosed and relapsed/refractory (R/R) AML [11,12,13,14]. Though pediatric experience with PD-1 inhibitors is limited in myeloid malignancies, nivolumab was shown to be well tolerated in 85 children and young adults with a variety of cancers in a Children’s Oncology Group (COG) trial (ADVL1412) [15]. While PD-1 inhibition is an exciting target in pediatric tumors, including AML, some cancers can evade this mechanism of action through the methylation of key transcription factors [16]. The reactivation of DNA methylation-silenced endogenous retroviruses via viral mimicry by DNA methylation inhibitors can stimulate an immune response and potentially convert non-immune-responsive tumors (*immune cold*) to immune-responsive ones (*immune hot*) [17,18]. The consequence of viral mimicry could have a potential synergistic effect via a combination treatment of PD inhibitors with epigenetic modifiers [19,20]. In addition, aberrant DNA methylation has been shown to be associated with development, relapse, and drug resistance in AML; the DNA methylation inhibitors, including decitabine (5-Aza-CdR) and azacitidine (5-Aza-CR), have been shown to increase patient survival [21,22,23,24]. Two previous Therapeutic Advances in Childhood Leukemia & Lymphoma (TACL) trials T2011-002 (NCT01861002) and T2016-003 (NCT03263936) showed tolerability and favorable responses to hypomethylating agents (HMAs) in combination with chemotherapy in pediatric patients with AML [25,26]. Hypomethylating agents upregulate the expression of the inhibitory checkpoint PD-1 on T cells, which, if left unchecked, promotes the exhaustion of tumor-specific T cells [20]. Hence, pretreatment or epigenetic priming with HMAs like azacitidine could lead to increased sensitivity to a PD-1 inhibitor [27]. Ultimately, the combination of a PD-1 inhibitor and HMA is rational in relapsed pediatric AML. Here, we report the results of a multi-institutional phase I/II study conducted by the TACL consortium (http://www.clinicaltrials.gov NCT03825367, accessed on 21 January 2024), which is the first to test nivolumab in combination with 5-azacitidine in pediatric patients with AML.

## 2. Methods

### 2.1. Study Design and Eligibility

This multicenter, open-label, single-arm, dose confirmation and cohort-expansion phase I/II trial was conducted through the TACL consortium at 21 centers in the USA (NCT03825367). This study was approved by the institutional review boards of participating institutions, and informed consent was obtained from all enrolled patients or their legal representatives. Eligible patients were aged 1 to 30 years, with AML with ≥5% blasts in the bone marrow or two serial marrows demonstrating stable or rising minimal residual disease (MRD) ≥ 0.1%, with or without extramedullary disease (EMD). Patients must have had a ≥1st relapse or refractory disease after original diagnosis following two induction attempts. Patients who experienced relapse after allogeneic hematopoietic stem cell transplantation (HSCT) were eligible, provided they had no evidence of active graft-versus-host-disease (GVHD) and were at least 100 days post-HSCT. Patients with Down’s syndrome (DS) were eligible on a separate stratum. Detailed eligibility criteria are listed in Table S1. 

Based on a 3 + 3 phase I design, nivolumab was given on day 1 at dose level 1 (3 mg/kg/day), with possible de-escalation to dose level 0 (1 mg/kg/day), along with 5-azacitidine (75 mg/m^2^) on days 1–7 (Table 1). After the “epigenetic priming,” a second dose of nivolumab was given on day 15 to enhance the checkpoint blockade on the regenerating CD4+/CD8+ T cells. Patients could receive two such cycles unless they had progressive disease (PD), as defined below. Primary objectives of the phase I portion were to establish a recommended phase II dose (RP2D) of nivolumab in combination with 5-azacitidine and to assess clinical activity in patients with relapsed/refractory (R/R) AML and ≥5% bone marrow (BM) blasts at study entry.

Additional patients were planned to be enrolled into phase II to accrue up to 20 response-evaluable patients at the RP2D. During phase II, there was one interim analysis planned after the twelfth evaluable patient completed cycle 1. A conditional power analysis was planned to be performed at that time. If, given the number of responses observed in the first 12 patients, the probability of rejecting the null hypothesis is less than 0.20 under the alternative where the odds ratio is 3.25, the study may be terminated for lack of sufficient efficacy. The conditional probability was estimated via simulation.

### 2.2. Toxicity Evaluation

Toxicity was graded according to the NCI Common Toxicity Criteria (CTC) version 5.0. Dose-limiting toxicity (DLT) events related to nivolumab were evaluated during cycle 1. Any patient who received fewer than 2 doses of nivolumab or fewer than 5 doses of azacitidine for reasons unequivocally unrelated to treatment toxicity, or who started subsequent anti-cancer therapy before the required observation times specific in the DLT definition, were considered not evaluable for DLT and were replaced. Hematological DLT was defined as failure to recover a peripheral blood (PB) absolute neutrophil count (ANC) > 500/μL and platelet count > 50,000/μL due to documented bone marrow hypoplasia (cellularity < 10%) by day 63 without evidence of active disease or active infection. Non-hematological DLT was defined as any grade 3 or higher non-immune related, non-hematologic toxicity that occurred after the first dose of nivolumab and was at least possibly attributable to nivolumab and prevented the patient from receiving subsequent therapy by day 63. 

Checkpoint inhibition is associated with a unique spectrum of side effects termed immune-related adverse events (irAEs). Dose-limiting toxicities for irAEs were defined as grade 2 or higher and included dermatologic, gastrointestinal, hepatic, endocrine, and autoimmunity events. Guidance for dose modifications and management of irAEs was provided in the protocol. 

### 2.3. Response Evaluation

Patients with >5% blasts in marrow at study entry who received at least 2 doses of nivolumab and at least 3 doses of azacitidine, or who received fewer doses for reasons related to toxicity or disease recurrence/progression, were considered evaluable for response. A bone marrow aspiration and/or biopsy to assess remission status was performed on day 29–36 when blood counts recovered. Complete response (CR) was defined as M1 marrow (<5% blasts) with no evidence of circulating blasts or EMD with recovery of PB counts (ANC > 500/μL and platelet count > 50,000/μL). Complete response without platelet recovery (CRp) was defined as M1 marrow with no evidence of circulating blasts or EMD in addition to recovery of ANC > 500/μL but insufficient recovery of a platelet count > 50,000/μL. Complete response with incomplete recovery (CRi) was defined as M1 marrow with no evidence of circulating blasts or EMD but with an ANC < 500/μL and/or a platelet count < 50,000/μL. CR with MRD negative was defined as M1 marrow and MRD ≤ 0.1% by central flow cytometry, with no evidence of circulating blasts or EMD and recovery of PB counts. Partial response (PR) was only assessed in patients who entered the study with >20% blasts in the marrow and was defined as complete disappearance of circulating blasts and a decrease of at least 50% of blasts in the bone marrow with >5% and <20% blasts by morphology and recovery of PB counts. Stable disease (SD) was defined as not meeting the criteria for CR, CRp, CRi, PR, or disease progression. Progressive disease (PD) was defined as an increase of at least 50% in the absolute number of bone marrow or circulating leukemic cells, development of new sites of EMD, and/or other laboratory or clinical evidence of progression of disease. MRD was measured by flow cytometry at the University of Washington Hematopathology Laboratory, Seattle, WA, USA.

### 2.4. Correlative Studies

Participation in correlative studies was optional with signed informed consent. The optional studies included questionnaires for quality of life (QoL) assessment and paired PB and bone marrow aspirate (BMA) samples for biologic correlatives.

#### 2.4.1. Quality of Life Assessment

Questionnaires utilizing PedsQL (Version 3.0 Cancer Module and Fatigue Module) and SSPedi (Symptom Screening in Pediatric) were optional and were completed by patients at study entry and at end of cycles.

#### 2.4.2. Flow Cytometry for T-Cell Immunophenotype and Immunohistochemical (IHC) Analysis of PD-L1

For this, 10-color flow cytometry was performed as per established protocol on pre-therapy and post-therapy PB and BMA at time of response evaluation between days 29 and 36 of the cycle [28]. Immunohistochemical (IHC) stain for PD-L1 was performed using the PD-L1 IHC 22C3 pharmDx kit on the Dako ASL48 platform according to manufacturer recommendations (Ventana Medical Systems Inc., Oro Valley, AZ, USA, (2017) VENTANA PD-L1 (SP263) assay) [29].

#### 2.4.3. DNA Methylation

Comprehensive DNA methylation profiling of PB and BMA was performed using the Illumina MethylationEPIC (EPIC) to interrogate DNA demethylation in PB and/or BMA specimens after the combination treatment, especially after azacitidine. The beta (β) value represents the DNA methylation score for each data point and is calculated as (M/(M + U)), in which M and U refer to the mean methylated and unmethylated probe signal intensities, respectively. Beta values range from 0 to 1, with 0 indicating an unmethylated locus and 1 indicating a fully methylated locus. Measurements in which the fluorescent intensity is not statistically significantly above background signal (detection *p* value > 0.05) as well as non-specific probes and those on the X- and Y-chromosomes were removed from the data set. 

## 3. Statistical Methods

The primary phase I endpoint was DLT assessment during cycle 1. Patients who received fewer than two doses of nivolumab or fewer than five doses of azacitidine for reasons unequivocally unrelated to treatment toxicity, or who started subsequent anti-cancer therapy before the required observation times specified in the DLT definition, were considered not evaluable for DLT and were replaced. Any patient who had received at least one dose of nivolumab was evaluable for toxicity. 

The primary phase II endpoint was the achievement of CR, CRp, and CRi at the end of cycle 1 for patients treated at the RP2D. Patients who received at least two doses of nivolumab and at least three doses of azacitidine, or who received fewer doses for reasons related to toxicity or disease recurrence/progression, were considered evaluable for response. 

Secondary objectives were to characterize the toxicities of this combination and to assess clinical activity in patients with R/R AML and <5% blasts at study entry. Secondary endpoints also included the assessment of toxicity profiles and achievement of undetectable MRD status, PedsQL (Version 3.0 Cancer Module and Fatigue Module) and SSPedi measures of QoL, PD-1 occupancy, PD-L1 expression, measure of T-cell activation, expression of immunoregulatory pathway genes, and measures of DNA methylation. 

Numeric data were summarized with median and range, while categorical data were summarized with the number and percentage of patients. Toxicity data were reported only for grade 3 or above, separately for course 1 and course 2 and for all attributions and those attributable to nivolumab. Patients with DS were to be enrolled in a separate secondary stratum with primarily descriptive analyses. Overall survival (OS) was defined from the start of protocol therapy to death from any cause. Patients who were alive were censored at the time of last follow-up in July 2023. OS was summarized using Kaplan–Meier estimates with standard errors estimated via the Greenwood formula. All analyses were performed using Stata version 17 (StataCorp, College Station, TX, USA).

## 4. Results

### 4.1. Patient Characteristics

Thirteen patients were enrolled (eight patients in the phase I portion from December 2019 to December 2020 and five patients in the phase II portion until September 2022), with a median age at enrollment of 13.7 years (range, 1.8–20.6) (Table 2). No patient with DS was enrolled. Eleven of the thirteen patients (85%) had > two prior treatment failures, with six patients (46%) never achieving a prior CR and seven patients experiencing a CR < 12 months (median prior regimens = 4, range = 1–9). The majority of patients had high disease burden (median BM blasts 78% and 40%, in phase I and phase II, respectively) (Table 2). Four patients had a history of HSCT at a median of 6 months (3–22 months) prior to the study treatment. Cytogenetics or molecular subgroup data for patients were not available as they were not required for enrollment in the study. 

### 4.2. Toxicity

Eleven of the thirteen patients were evaluable for DLT (received at least two doses of nivolumab) (Table 2). There were no DLTs noted in these 11 patients, and no patient discontinued therapy due to AEs (Table 2 and Table S2). Two patients were not evaluable for DLT, as both only received one dose of nivolumab, one patient due to a parental decision to discontinue protocol therapy mid-cycle, and one patient due to PD. Of note, no irAEs or graft-versus-host disease were reported in any patients, including those with prior history of HSCT. Grade 3–4 AEs were primarily hematological (Table S2). Febrile neutropenia in five patients was the most common ≥ grade 3 AE. One patient developed grade 5 cardiac arrest due to PD, deemed unrelated to nivolumab. 

### 4.3. Response

Twelve of the thirteen patients were evaluable for response. One patient withdrew after the first dose of protocol therapy due to parental decision and, hence, was not evaluable for response. The RP2D was established at dose level 1 (3 mg/kg/dose). Of the twelve evaluable patients treated at the RP2D, four (33%) had SD and none achieved a CR after the first cycle of treatment (Table S3). Two patients with SD received a second cycle and maintained SD, proceeding to HSCT after alternative disease-directed therapy. On follow-up of the 13 patients, 5 went on to receive palliative care, 6 patients received additional chemotherapy regimens, and 2 patients underwent HSCT. At 6 months post last patient enrollment, 12/13 were deceased. The 6-month overall survival estimate is 38.5% (95% confidence interval 14.1–62.8%) (Figure S1).

Based on 10,000 simulations, the probability of rejecting the null hypothesis (i.e., the conditional power) was close to 0, under the alternative where the odds ratio was 3.25 and the one-sided Type I error rate was 0.15. The futility boundary of 0.20 was crossed, indicating the likelihood that the study, if continued, would conclude with a positive result was very small; hence, the study accrual was terminated. 

### 4.4. Quality of Life

Six patients participated in the PedsQL and SSPedi questionnaires, with three patients providing their answers before and after the first cycle of therapy (Figure S2). All three showed a decrease in total cancer module score and fatigue score after one cycle of therapy, across all eight domains of the cancer module (pain and hurt, nausea, procedural anxiety, treatment anxiety, worry, cognitive problems, perceived physical appearance, and communication) and all three domains of the fatigue module (general fatigue, sleep/rest fatigue, and cognitive fatigue). A decrease in total score corresponded to an improvement in quality-of-life domains.

### 4.5. Correlative Biology 

Flow cytometry for T-cell immunophenotype and immunohistochemical (IHC) analysis of PD-L1

**Figure 1:**
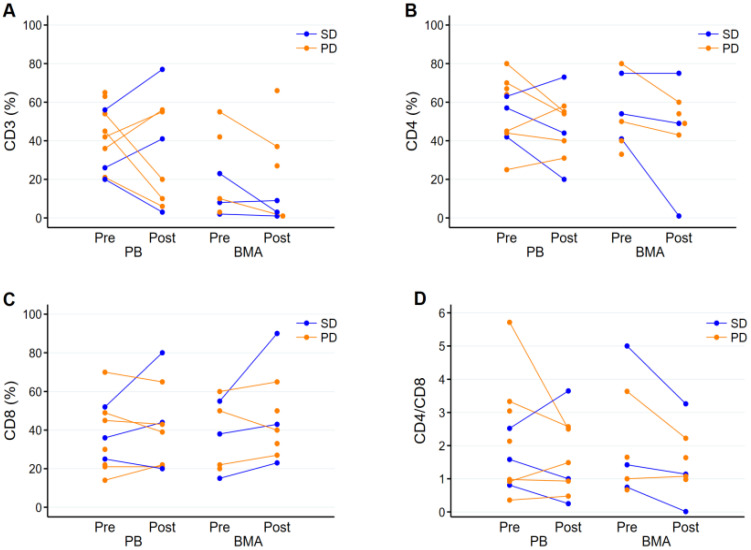
Pre- and post-treatment T-cell subsets of total leukocytes measured in peripheral blood (PB) and bone marrow aspirate (BMA) samples. (**A**): Percentages of pre- and post-treatment CD3+ T-cells. Most patients had elevated baseline levels in BMA (normal range 57-89% in PB and 3–8% in BMA). (**B**) Percentages of CD4+ T cells elevated in most patients with a trend of decrease post-treatment with PD-1 inhibitor nivolumab, more noticeable in patients with stable disease (SD, blue) compared to patients with progressive disease (PD, orange) (normal range 30–60% in PB). (**C**) Percentages of CD8+ T cells elevated in most patients at baseline with further increase post-treatment with PD-1 inhibitor nivolumab, noticeable in patients with SD (normal range 19–43% in PB). (**D**) Ratio of CD4+/CD8+ T cells shows reduction in the ratio post-treatment with PD-1 inhibitor nivolumab in most patients, especially in patients with SD, suggesting effect of checkpoint inhibition.

For analysis of checkpoint inhibition, 10 of the 13 patients consented to provide samples. Eight patients had paired pre- and post-treatment PB, five had paired BMA, while three opted out for providing post-treatment BMA samples. In the patients with paired samples, median pre-treatment CD3+ T cells represented 39% (range: 20–56%) and 10% (2–55%) of total leukocytes in PB and BMA, respectively, by flow cytometry (Figure 1A). In the same patients, median post-treatment CD3+ T cells represented 30.5% (3–77%) and 3% (1–37%) in PB and BMA samples, respectively (Figure 1A). The median pre-treatment CD4+ T cells were 51% (25–80%) and 54% (41–80%), and post-treatment was 49% (20–73%) and 49% (1–75%) in PB and BMA, respectively (Figure 1B). The median pre-treatment CD8+ T cells were 40.5% (14–70%) and 44% (15–60%), and post-treatment was 41% (20–80%) and 41.5% (23–90%) in PB and BMA, respectively. There was a trend towards an increase in CD8+ T cells post-treatment. (Figure 1C). The median pre-treatment ratios of CD4+ and CD8+ T cells were 1.3 (0.4–5.7) and 1.4 (0.7–5), and, for post-treatment, there was a reduction in the ratio to 1.2 (0.3–3.7) and 1.1 (0–3.3) in PB and BMA, respectively (Figure 1D). Only rare NK cells were noted on all the samples. 

IHC analysis of PD-L1 on pre- and post-treatment tumor specimens (BM clot section) where available was performed to study the role of intratumoral modulation of the PD-1-PD-L1 pathway. The IHC stain was performed on seven samples (five patients), and only two patients had both pre- and post-BM samples adequate for staining, one each with SD and PD. PD-L1 staining was positive in both pre- and post-BM samples in the patient with SD, while it was negative in both samples in the patient with PD (Figure S3).

### 4.6. DNA Methylation

**Figure 2:**
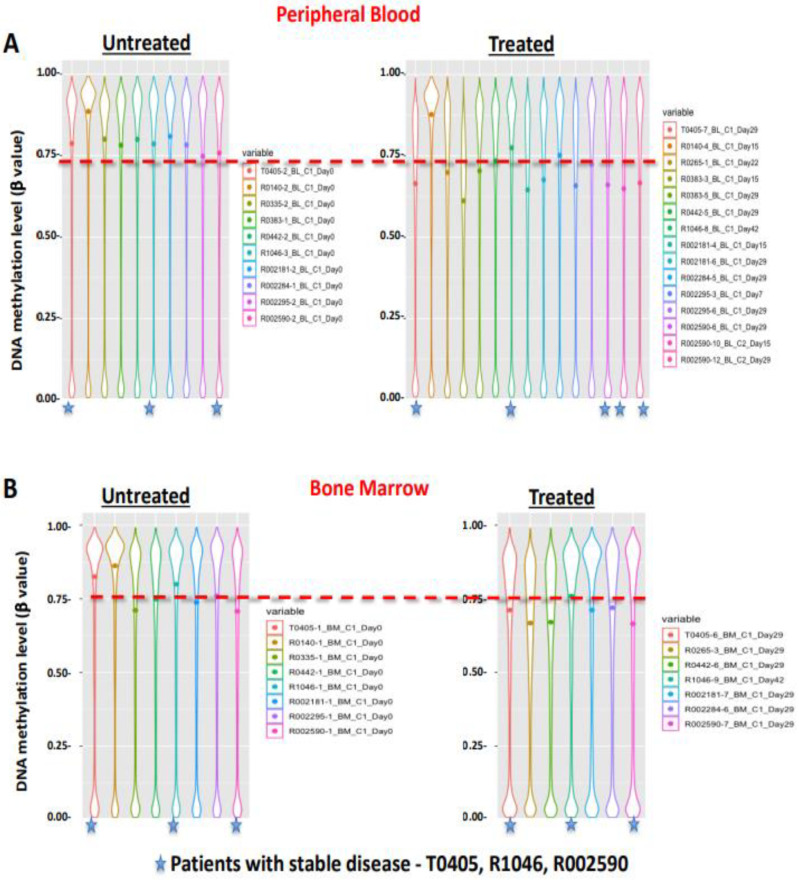
Violin plot showing the global DNA methylation level of DNA from patients before and after treatment in (**A**) peripheral blood and (**B**) bone marrow. The median methylation values for each subject are shown as a dot. Subjects with stable disease (T0405, R1046, R002590) show reduction in methylation post-treatment indicated with a star.

**Figure 3:**
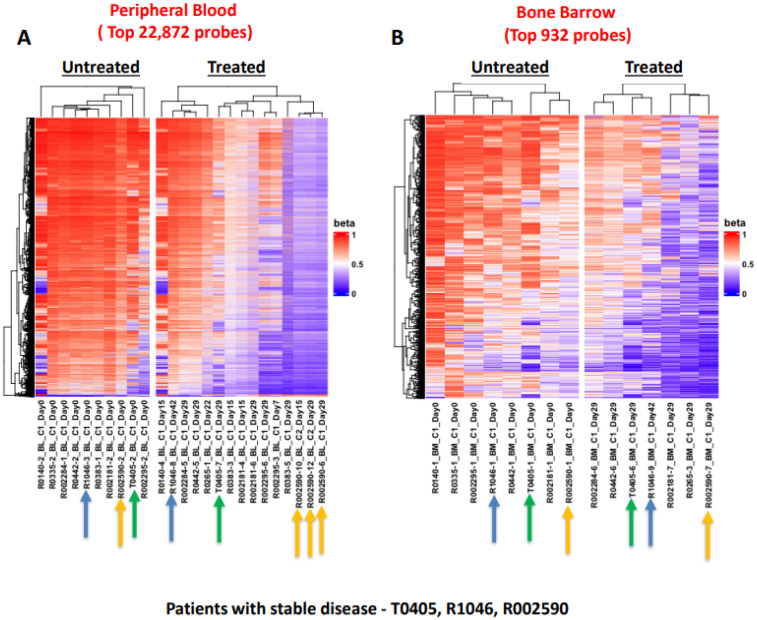
Supervised hierarchical cluster of the 22,872 probes in peripheral blood (**A**), and 937 probes in bone marrow aspiration (**B**) demonstrate differentially methylated probes in samples pretreatment and post treatment with hypomethylating agents. Increase in demethylation is noticeable in most samples after treatment, especially in subjects with stable disease (T0405, R1046, R002590) indicated with corresponding matching color arrows.

**Figure 4:**
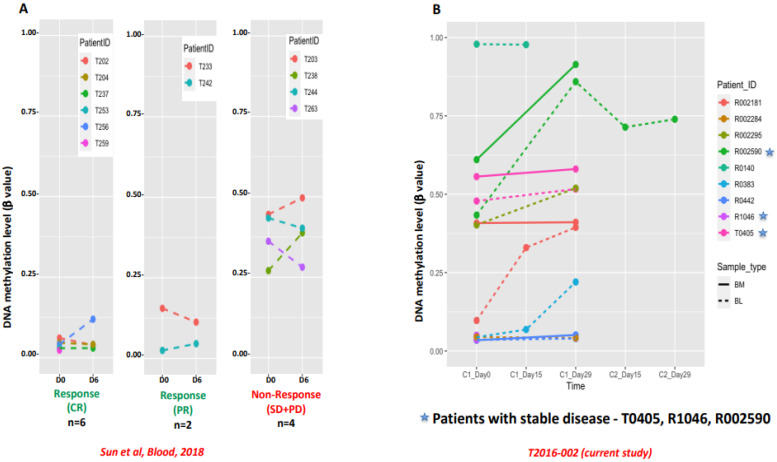
(**A**) DNA methylation level at probe (cg11598005) before treatment (day 0) and after treatment (day 6). Response (CR: *n* = 6, PR: *n* = 2) and non-response (SD + PD: *n* = 4) from Sun et al., Blood, 2018 [25]. (**B**) DNA methylation level at probe (cg11598005) in current study before treatment (cycle 1, day 0) and after treatment (cycle1 day 15, cycle 1 day 29, cycle 2 day 15 and cycle 2 day 29). Majority of patients had elevated levels of DNA methylation at baseline. Patients with stable disease (R002950, R 1046 and T0405) are indicated with a star. R002950 had samples post cycles 1 and 2, which showed high DNA methylation at baseline, which decreased post first cycle and remained stable post second cycle. R1046 had low level at baseline, which remained low post 1st cycle. T0405 had high level at baseline, which remained stable post 1st cycle. DNA methylation in patients with progressive disease increased post therapy. Solid line represents the DNA from bone marrow sample and dashed line DNA from peripheral blood sample (BL).

For analysis of DNA methylation, 11 of the 13 patients opted to provide samples, which included 3 of the 4 patients who had SD. DNA methylation changes before and after treatment were analyzed in PB and BMA samples. By analyzing the global methylation level, baseline high DNA methylation was observed in untreated PB and BMA samples, which decreased after HMA treatment; these changes were more effectively demonstrated in PB samples and in samples of the three patients who had SD (Figure 2). Welch’s *t*-test was applied on the filtered 22,872 probes in PB samples (Figure 3A) and 923 probes for BMA samples (Figure 3B), which demonstrated an increase in demethylation post-treatment. Though we observed a trend of demethylation post-treatment with HMA in most patients and more noticeable in patients with SD, since no patient had a CR or PR, it was challenging to correlate the DNA methylation data with the clinical outcome (Figure 3). In this study, we analyzed the impact of the DNA methylation marker located in a region of the gene *FDFT1* that was shown to be associated with response on a previous TACL consortium study with azacitidine (T2011-002, *NCT01861002*) (Figure 4A) [25]. This DNA methylation marker (cg11598005) was highly methylated in PB and BMA and further increased after treatment in four out of seven in PB and two out of four in BMA (Figure 4B), all patients who had PD. Of the three patients with SD, two had no change in this marker post-treatment, while one patient who opted to provide samples during cycle 2 had a reduction in the level of this methylation marker after the first cycle of therapy, which remained stable post cycle 2 (subject R002590, Figure 4B). 

## 5. Discussion

This trial demonstrated that the combination of nivolumab and azacitidine is well tolerated and has an acceptable safety profile in pediatric patients with R/R AML. No immune-related toxicities or DLTs were observed. Combinations of HMAs like azacitidine with nivolumab have been associated with an improvement in survival in adults with R/R AML (median prior therapies for AML = 2) [11]. Compared to the adult cohort, pediatric patients in our trial were heavily pre-treated (median prior therapies = 4, range = 1–9) and had higher disease burden. Clinical trials with checkpoint inhibitors and HMAs in adults with AML and myelodysplastic syndromes have demonstrated prolonged survival with multiple cycles, which was not possible to demonstrate in our trial since the majority of patients only received one cycle before proceeding to alternative therapy or palliative care [14,30]. Although none of the patients achieved a CR, the only two patients with lower disease burden (M2 marrow) at study entry had SD. Pediatric AML is a highly proliferative disease, with notable differences compared to adult AML, specifically differences in genetic landscapes, as demonstrated by the Therapeutically Applicable Research to Generate Effective Treatments (TARGET) AML project [31]. Although no patient achieved a CR, some patients appeared to derive clinical benefit, as demonstrated by the four (33%) patients who had SD as well as improvements in QoL scores in the patients who took the surveys. 

None of the patients had MRD-level disease at study entry in contrast to elderly patients with AML who have smoldering MDS/AML, for whom the combination showed efficacy in previous reports [25]. Nivolumab was effective in eradicating MRD-level disease and extended duration of remission in high-risk AML patients not eligible for allogeneic HSCT in adults, suggesting that further trials should explore this strategy in pediatric patients with lower disease burden [32]. The use of nivolumab could also prove to be effective in post-HSCT relapse settings to maintain remission by improving the T-cell effector functions as well as the graft-versus-leukemia effect [33]. Careful planning of conditioning regimens and post-HSCT GVHD prophylaxis with cyclophosphamide can be effective as strategies to minimize irAE and GVHD [33,34,35,36]. In addition, this regimen could be studied as maintenance therapy in pediatric patients, similar to recent studies in adults demonstrating the benefit of azacitidine, leading to the U.S. FDA’s approval of oral azacitidine for maintenance therapy in adults with AML in first remission [37,38,39]. The REMAIN clinical trial in adults is an ongoing, randomized phase II study of nivolumab for the eradication of MRD and prevention of relapse in high-risk AML in remission (NCT02275533). The combination of azacitidine and nivolumab may also be potentially beneficial in the extramedullary relapse of AML [40]

While few studies have analyzed the immunological markers in patients receiving PD-1 inhibitors, some have demonstrated higher CD3+ and CD8+ T cells in pre-therapy PB and BMA to be a marker of response to immunotherapy in adult AML patients [7]. Patients with advanced metastatic tumors who responded to PD-1 inhibitors had increased levels of CD3+ and CD8+ T cells in the PB at baseline. Opposing effects on CD4^+^ and CD8^+^ cell populations have been observed after nivolumab therapy, with CD4^+^ cell levels declining and the proportion of Treg cells and CD8^+^ T cells increasing [41,42]. In three of the four patients with SD, where pre- and post-samples were available to evaluate, there was a trend toward an increase in CD8+ T cells after nivolumab therapy and a reduction in the CD4+/CD8+ ratio, suggesting an in vivo effect of PD-1 inhibition (Figure 1). With the limited number of patients and doses of nivolumab, and the absence of clinical responses, it is difficult to draw definite correlations of this trend with response outcomes. 

The majority of patient samples (6 out of 10 in PB and 7 out of 7 in BMA in Figure 3) exhibited demethylation after treatment with the DNA methylation inhibitor azacitidine. This trend was more noticeable in patients with SD. Furthermore, we noted that a DNA methylation marker (cg11598005) associated with response on a previous TACL study with azacitidine (T2011-002) might serve not only as a predictor of poor response to the treatment but also as a means of monitoring treatment [25]. This marker showed increased DNA methylation in samples of patients with PD after the combination treatment. 

The limitations of this study were the small number of patients, with multiple previous cytotoxic chemotherapy attempts, and heterogenous AML subtypes. The study design only permitted two cycles of protocol therapy; however, additional cycles may have provided clinical benefit for patients with stable disease.

## 6. Conclusions

Nivolumab in combination with 5-azacitidine had an acceptable safety profile and was well tolerated in heavily pre-treated pediatric patients with R/R AML at the RP2D (3 mg/kg/dose). This combination therapy could be administered safely on an outpatient basis and improved the QoL of the participants. Correlative biology studies revealed an increase in CD3+ T cells and CD8+ T cells and a trend of increase in CD8+ T cells and CD4+/CD8+ after nivolumab treatment. Heavily pre-treated patients with R/R AML exhibited higher levels of methylation, and treatment with HMAs showed a trend for a reduction in methylation in patients with SD. Additionally, this study further validated the use of the DNA methylation marker (cg11598005) to predict response and monitor the effectiveness of HMA treatment. This study was closed to accrual following a futility analysis based on the study design. Future studies should evaluate the efficacy of this combination in other subsets of pediatric patients with AML, such as those with lower disease burden, extramedullary relapse, and as a means to maintain remission post chemotherapy or post HSCT in pediatric patients at high risk of relapse. 

## Figures and Tables

**Table 1:** Treatment course.

Days	-5-7	1	2	3	4	5	6	7	15	29-36
IT cytarabine	X									
Nivolumab		X							X	
5-azacytidine		X	X	X	X	X	X	X		
Nivolumab dose	3 mg/kg, maximum single dose not to exceed 240 mg If 3 mg/kg is not tolerated in combination with 5-azacytidine, the dose will be stepped down to 1 mg/kg									
5-azacytidine	75 mg/m^2^ subcutaneously or intravenously									
IT Cytarabine	30 mg for patients age 1–1.99 years 50 mg for patients age 2–2.99 years 70 mg for patients ≥3 years of age									

Patients who achieved a CR, CRp, CRi, PR or SD could receive 2 courses of therapy in the absence of progressive disease or DLT. Abbreviations: IT intrathecal, CR complete response, CRp complete response without platelet recovery, CRi complete response with incomplete recovery, PR partial response, SD stable disease, DLT dose limiting toxicity.

**Table 2:** Patient characteristics and outcomes.

Variable		Study Phase		
		Phase I (n = 8)	Phase II (n = 5)	Total (n = 13)
**Median Age at enrollment, in years (range)**		13.3 (2.7, 19.9)	12 (1.8, 20.6)	13.7 (1.8, 20.6)
**Sex**	Male	5 (62%)	2 (40%)	7 (54%)
	Female	3 (38%)	3 (60%)	6 (46%)
**Race**	White	4 (50%)	2 (40%)	6 (46%)
	Black or African American	0 (0%)	1 (20%)	1 (8%)
	Native Hawaiian/Pac. Isl.	1 (12%)	0 (0%)	1 (8%)
	Not Reported	3 (38%)	2 (40%)	5 (38%)
**Ethnicity**	Hispanic/Latino	4 (50%)	2 (40%)	6 (46%)
	Non-Hispanic/Latino	4 (50%)	3 (60%)	7 (54%)
**Median number of prior therapy attempts**		4.5 (2, 9)	2 (1, 7)	4 (1, 9)
**Prior HSCT**	No	6 (75%)	3 (60%)	9 (69%)
	Yes	2 (25%)	2 (40%)	4 (31%)
**Prior Failure**	1	0 (0%)	2 (40%)	2 (15%)
	2+	8 (100%)	3 (60%)	11 (85%)
**Prior Response**	Never achieved CR	4 (50%)	2 (40%)	6 (46%)
	CR for ≤12 Months	4 (50%)	1 (20%)	5 (39%)
	CR for >12 Months	0 (0%)	2 (40%)	2 (15%)
**Disease burden on study**				
**Median (range) % bone marrow blasts**		78 (12, 95)	40 (7, 91)	70 (7, 95)
**Presence of** **peripheral blast**	No	4 (50%)	2 (40%)	6 (46%)
	Yes	4 (50%)	3 (60%)	7 (54%)
**CNS disease**	Negative	8 (100%)	5 (100%)	13 (100%)
**Study Outcomes**				
**DLT** †	No	6 (100%)	5 (100%)	11 (100%)
**Response** †	SD	2 (29%)	2 (40%)	4 (33%)
	PD	5 (71%)	3 (60%)	8 (67%)
**Vital status**	Alive	0 (0%)	1 (20%)	1 (8%)
	Deceased	8 (100%)	4 (80%)	13 (92%)

Abbreviations: n number, DLT dose limiting toxicity, PD progressive disease, SD stable disease, CNS central nervous system. † Excluding patients not evaluable for DLT or response. Note: There were no patients with Down syndrome enrolled in this study.

## Data Availability

De-identified participant data that support the findings of this article will be shared upon approved written request at 2 years after this article publication for 7 years from the publication date. These data will be available to researchers who provide a methodologically sound proposal for the purposes of achieving specific aims outlined in that proposal. Proposals should be directed to TACL Consortium Central Inbox TACL@chla.usc.edu (Attention: TACL Consortium Administrative Director, Erika Shin-Kashiyama, JD) and will be reviewed by the TACL Steering and Prioritization Committee. Requests to access data to undertake hypothesis-driven research will not be unreasonably withheld. To gain access, data requesters will need to sign a data access agreement and to confirm that data will only be used for the agreed purpose for which access was granted.

## Supplementary Materials

The following supporting information can be downloaded at: https://www.mdpi.com/article/10.3390/cancers16030496/s1, Figure S1: Overall Survival; Figure S2: Quality of life Results; Figure S3: immunohistochemistry for PD-1 staining; Table S1: Inclusion and Exclusion Eligibility Criteria; Table S2: Grade 3 or higher adverse events (AEs); Table S3: Interim Data for Futility Analysis.
