# Supplementary material for: Nivolumab Plus 5-Azacitidine in Pediatric Relapsed/Refractory Acute Myeloid Leukemia (AML): Phase I/II Trial Results from the Therapeutic Advances in Childhood Leukemia and Lymphoma (TACL) Consortium
Source: Cancers (Basel). 2024 Jan 24;16(3):496. doi: 10.3390/cancers16030496 (PMC10854518; doi:10.3390/cancers16030496)

Supplementary Table S1: Inclusion and Exclusion Eligibility Criteria

**Age**<br>≥ 1 and ≤30 years of age
**Diagnosis**<br>**Relapsed or refractory AML with ≥5% blasts (by morphology) in the bone marrow**<br>-1st or greater relapse, OR<br>-Failed to go into remission (i.e., refractory) after first or greater relapse, OR<br>-Failed to go into remission from original diagnosis after two or more induction attempts<br><br>**Relapsed or refractory AML with ≤ 5% blasts (by morphology) and MRD positive disease (M1/MRD+)**<br>-Two serial marrows demonstrating stable or rising MRD ≥ 0.1 % (i.e. not declining)
**CNS Status**<br>Patients must have CNS negative status (CNS1). No cranial irradiation is allowed during the protocol therapy
**Extramedullary Disease**<br>Patients with extramedullary disease other than CNS disease are eligible only if bone marrow disease is also present (i.e., combined relapse)
**Secondary AML**<br>Patients with secondary AML are eligible. Of note, these patients will be eligible even without prior therapy for AML.
**Down Syndrome**<br>Patients with Down Syndrome will be eligible and will be included as an observation cohort
**Other syndromes**<br>Patients with DNA fragility syndromes (such as Fanconi anemia, Bloom syndrome) are excluded
**Performance Level**<br>Karnofsky > 50% for patients > 16 years of age and Lansky > 50% for patients ≤ 16 years of age
**Prior Therapy**<br>Patients must have fully recovered from the acute toxic effects of all prior chemotherapy, immunotherapy, or radiotherapy prior to entering this study.<br><br>**Myelosuppressive chemotherapy**<br>a) **Prior chemotherapy** Patients must have fully recovered from the acute toxic effects of all prior chemotherapy, immunotherapy, or radiotherapy prior to entering this study. At least 14 days must have elapsed since the completion of the cytotoxic therapy, except Intrathecal chemotherapy.<br>b) **Cytoreduction with hydroxyurea** Hydroxyurea can be initiated and continued for up to 24 hours prior to the start of day 1 nivolumab and azacytidine. It is recommended to use hydroxyurea in patients with significant leukocytosis (WBC > 50,000/L) to control blast count before initiation of systemic protocol therapy.<br>c) **Hematopoietic stem cell transplant**: Patients who have experienced their relapse after a HSCT are eligible provided they have no evidence of active GVHD, no past history of grade 3 or greater GVHD, and are at least 100 days post-transplant at the time of enrollment. Patients should be off immune suppression for at least 2 weeks (excluding physiologic replacement steroids).<br>d) **Hematopoietic growth factors**: It must have been at least 7 days since the completion of therapy with GCSF or other growth factors at the time of enrollment. It must have been at least 14 days since the completion of therapy with pegfilgrastim (Neulasta®).<br>e) **Biologic (anti-neoplastic agent)**: At least 7 days after the last dose of a biologic agent. For agents that have known adverse events occurring beyond 7 days after administration, this period must be extended beyond the time during which adverse events are known to occur. The duration of this interval must be discussed with the study chair

**f)** **Monoclonal antibodies**: At least 3 half -lives of the antibody must have elapsed after the last dose of monoclonal antibody. (i.e., Gemtuzumab = 36 days)<br>**g)** **Immunotherapy**: At least 42 days after the completion of any type of immunotherapy, e.g., tumor vaccines or CAR T-cells.<br>**h)** **XRT**: XRT is prohibited during protocol therapy. No washout period is necessary for radiation given to non-CNS chloromas; ≥ 90 days must have elapsed if prior TBI or craniospinal XRT.
**Organ function**<br>• GFR ≥ 70ml/min/1.73m2 or normal serum creatinine<br>• Direct bilirubin < 1.5 x ULN for age and ALT < 5x ULN for age<br>• Shortening fraction of ≥ 27% or ejection fraction >50% by echocardiogram

*MRD will be determined by multiparameter flow cytometry using AML-associated phenotype markers, or real- time quantitative PCR for AML-associated genetic lesions

Abbreviations: AML acute myeloid leukemia, MRD minimal residual disease, WBC white blood cells, HSCT hematopoietic stem cell transplant, GVHD graft versus host disease, GCSF granulocyte colony-stimulating factor, CAR T-cells chimeric antigen receptor T cells, XRT radiation, TBI total body irradiation, ULN upper limit of normal

Supplementary Table S2 – Grade 3 or higher adverse events (AEs)

	Course 1 AEs regardless of attribution n (%)			Course 1 AEs attributable to Nivolumab n (%)			Course 2 AEs regardless of attribution n (%)			Course 2 AEs attributable to Nivolumab n (%)		
	Grade 3	Grade 4	Grade 5	Grade 3	Grade 4	Grade 5	Grade 3	Grade 4	Grade 5	Grade 3	Grade 4	Grade 5
**Non-Hematological**												
Febrile neutropenia	5 (38)	0 (0)	0 (0)	0 (0)	0 (0)	0 (0)	1 (50)	0 (0)	0 (0)	1 (50)	0 (0)	0 (0)
Cardiac arrest	0 (0)	0 (0)	1 (8)	0 (0)	0 (0)	0 (0)	0 (0)	0 (0)	0 (0)	0 (0)	0 (0)	0 (0)
Pericardial effusion	0 (0)	1 (8)	0 (0)	0 (0)	0 (0)	0 (0)	0 (0)	0 (0)	0 (0)	0 (0)	0 (0)	0 (0)
Nausea	1 (8)	0 (0)	0 (0)	0 (0)	0 (0)	0 (0)	0 (0)	0 (0)	0 (0)	0 (0)	0 (0)	0 (0)
Disease progression	0 (0)	0 (0)	2 (15)	0 (0)	0 (0)	0 (0)	0 (0)	0 (0)	0 (0)	0 (0)	0 (0)	0 (0)
Fatigue	1 (8)	0 (0)	0 (0)	0 (0)	0 (0)	0 (0)	0 (0)	0 (0)	0 (0)	0 (0)	0 (0)	0 (0)
Generalized edema	1 (8)	0 (0)	0 (0)	0 (0)	0 (0)	0 (0)	0 (0)	0 (0)	0 (0)	0 (0)	0 (0)	0 (0)
Catheter related infection	2 (15)	0 (0)	0 (0)	0 (0)	0 (0)	0 (0)	0 (0)	0 (0)	0 (0)	0 (0)	0 (0)	0 (0)
Gluteal Abscess	1 (8)	0 (0)	0 (0)	0 (0)	0 (0)	0 (0)	0 (0)	0 (0)	0 (0)	0 (0)	0 (0)	0 (0)
Lung infection	2 (15)	0 (0)	0 (0)	0 (0)	0 (0)	0 (0)	0 (0)	0 (0)	0 (0)	0 (0)	0 (0)	0 (0)
Sepsis	1 (8)	0 (0)	0 (0)	0 (0)	0 (0)	0 (0)	0 (0)	0 (0)	0 (0)	0 (0)	0 (0)	0 (0)
Alkaline phosphatase increased	1 (8)	0 (0)	0 (0)	0 (0)	0 (0)	0 (0)	0 (0)	0 (0)	0 (0)	0 (0)	0 (0)	0 (0)
Weight loss	1 (8)	0 (0)	0 (0)	0 (0)	0 (0)	0 (0)	0 (0)	0 (0)	0 (0)	0 (0)	0 (0)	0 (0)
Anorexia	1 (8)	0 (0)	0 (0)	0 (0)	0 (0)	0 (0)	0 (0)	0 (0)	0 (0)	0 (0)	0 (0)	0 (0)
Hypoalbuminemia	1 (8)	0 (0)	0 (0)	0 (0)	0 (0)	0 (0)	0 (0)	0 (0)	0 (0)	0 (0)	0 (0)	0 (0)
Hypocalcemia	1 (8)	0 (0)	0 (0)	0 (0)	0 (0)	0 (0)	0 (0)	0 (0)	0 (0)	0 (0)	0 (0)	0 (0)
Hypokalemia	2 (15)	0 (0)	0 (0)	0 (0)	0 (0)	0 (0)	0 (0)	0 (0)	0 (0)	0 (0)	0 (0)	0 (0)
Dyspnea	1 (8)	0 (0)	0 (0)	0 (0)	0 (0)	0 (0)	0 (0)	0 (0)	0 (0)	0 (0)	0 (0)	0 (0)
Epistaxis	1 (8)	0 (0)	0 (0)	0 (0)	0 (0)	0 (0)	0 (0)	0 (0)	0 (0)	0 (0)	0 (0)	0 (0)
Hypoxia	1 (8)	0 (0)	0 (0)	0 (0)	0 (0)	0 (0)	1 (50)	0 (0)	0 (0)	1 (50)	0 (0)	0 (0)
Pulmonary edema	1 (8)	0 (0)	0 (0)	0 (0)	0 (0)	0 (0)	0 (0)	0 (0)	0 (0)	0 (0)	0 (0)	0 (0)
**Hematological**												
Anemia	11 (85)	0 (0)	0 (0)	3 (23)	0 (0)	0 (0)	2 (100)	0 (0)	0 (0)	1 (50)	0 (0)	0 (0)
Lymphocyte count decreased	0 (0)	2 (15)	0 (0)	1 (8)	0 (0)	0 (0)	1 (50)	0 (0)	0 (0)	0 (0)	0 (0)	0 (0)
Neutrophil count decreased	0 (0)	5 (38)	0 (0)	0 (0)	2 (15)	0 (0)	0 (0)	1 (50)	0 (0)	0 (0)	1 (50)	0 (0)
Platelet count decreased	0 (0)	8 (62)	0 (0)	0 (0)	1 (8)	0 (0)	0 (0)	1 (50)	0 (0)	0 (0)	0 (0)	0 (0)
White blood cell decreased	0 (0)	7 (54)	0 (0)	1 (8)	0 (0)	0 (0)	0 (0)	1 (50)	0 (0)	0 (0)	1 (50)	0 (0)

Supplementary Table S3 – Interim Data for Futility Analysis

Stratification Factor		Interim Data	
Prior Response duration	Number of prior treatment attempts	Number of patients	Number of CRs
Never had a CR	1st induction failure	0	0
	2nd+ (re)induction failure	5	0
CR lasting < 12 months	1st induction failure	1	0
	2nd+ (re)induction failure	4	0
CR lasting 12+ months	1st induction failure	1	0
	2nd+ (re)induction failure	1	0
Total		12	0

Supplementary Figure S1 - Overall Survival

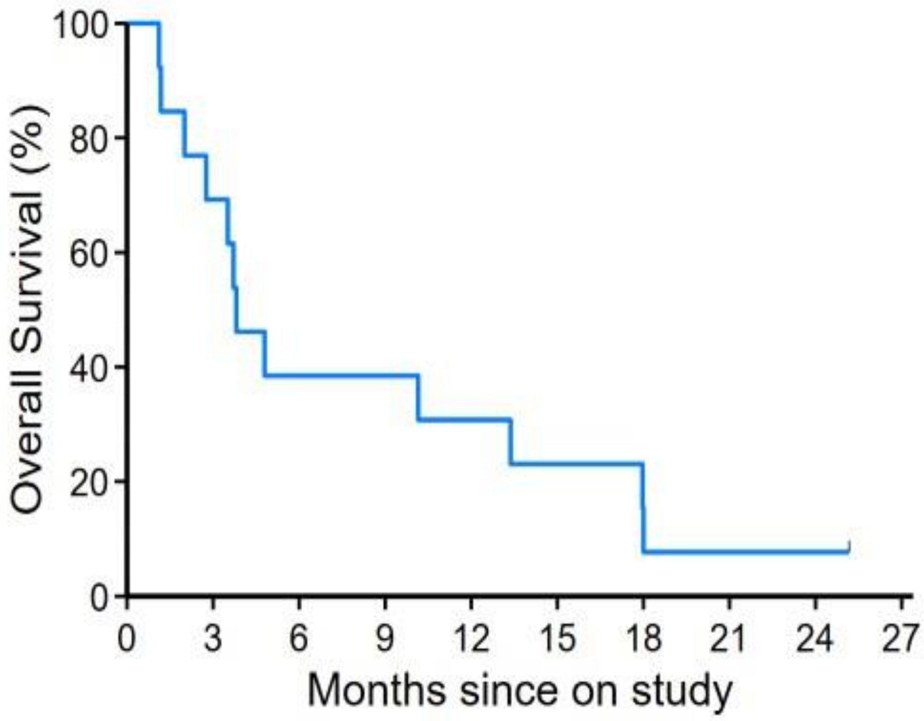

Supplementary Figure S2 – Quality of Life Results

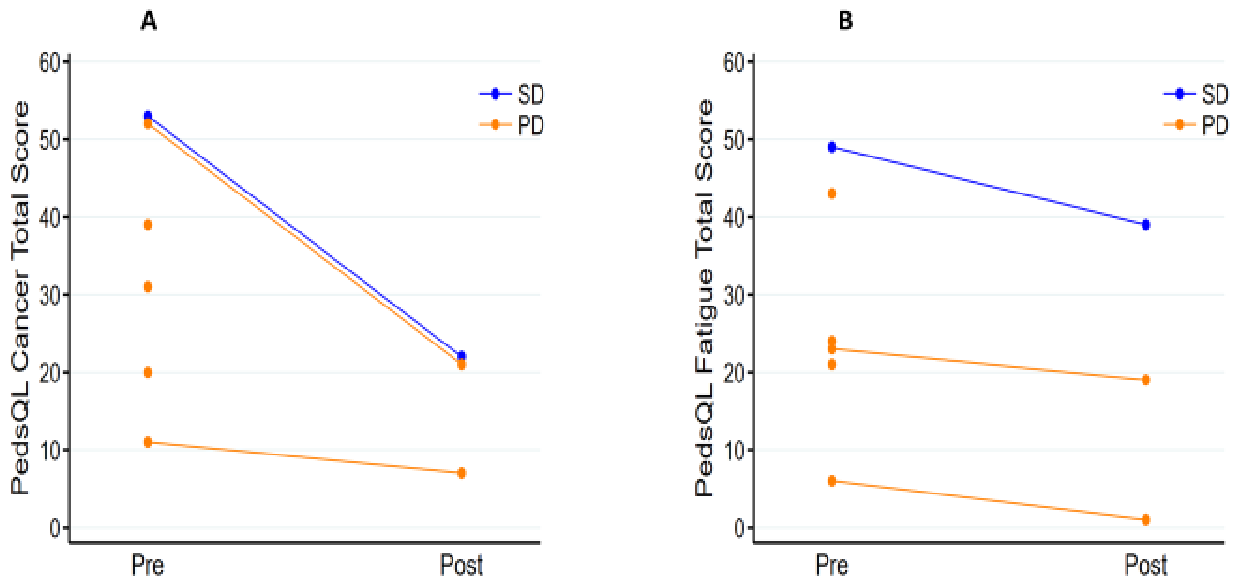

Supplementary Figure S3 – Immunohistochemistry for PD-1 staining

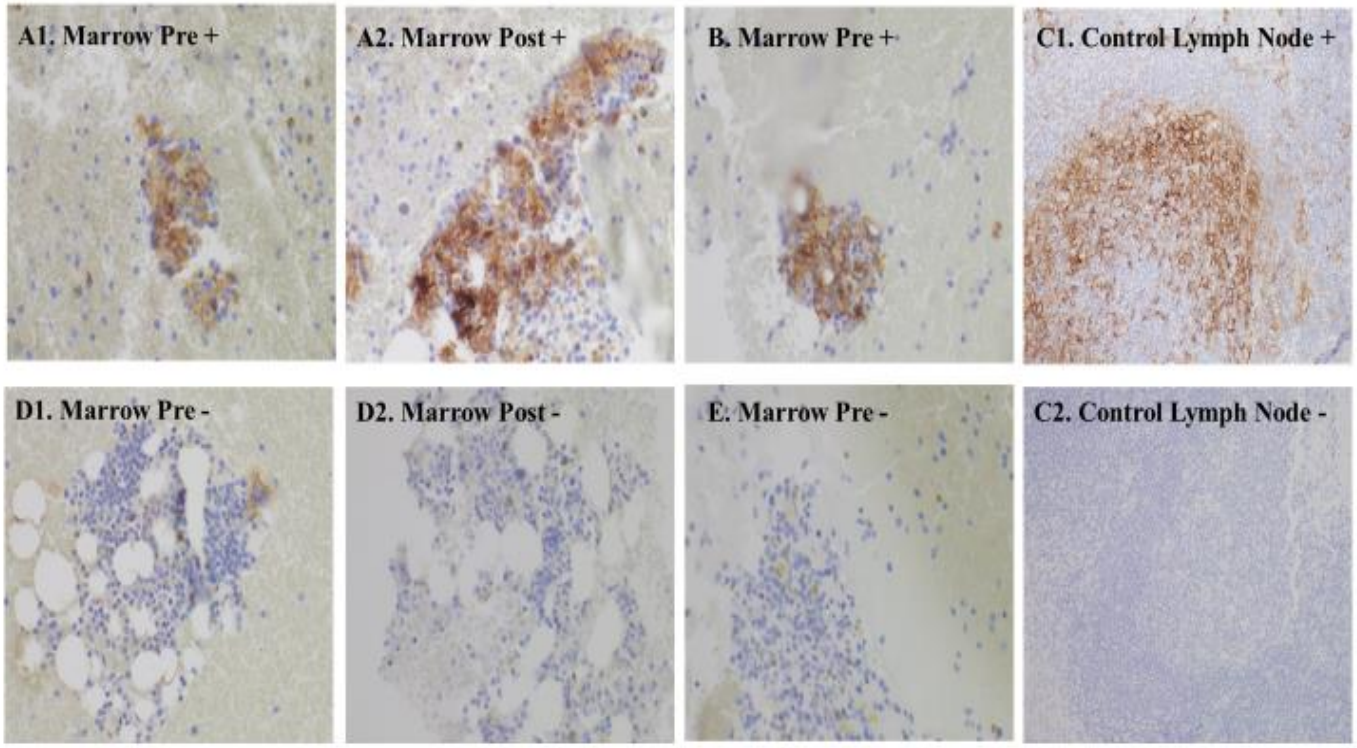

Supplement: Supplementary file 1 [file cancers-16-00496-s001.zip › cancers-2786527-supplementary.pdf]
